# Idiosyncrasy

**Published:** 1909-03-13

**Authors:** 


### Dietetics.

### IDIOSYNCRASY.

In matters of diet, as in most other things, the natural inclinations and tastes of the individual should receive due attention from the medical attendant. The practitioner should study his patient and not devote all his care to the case merely. And in doing so he will have to consider very carefully the wide question of individual idiosyncrasy.

It is only of late years that the full importance of idiosyncrasy in matters of diet has attracted the attention of the profession in the degree it deserves, and it is safe to assert that there are many instances in which it is even yet disregarded. Every practitioner knows that there is a vast amount of truth underlying the old dictum that one man's meat is another man's poison. The public at large has not yet grasped the full meaning of this fact. It still runs amok on diet fads; it still argues that because this or that thing has been found good in this or that case it must, therefore, be equally good in another case.

That certain foods affect certain individuals in a manner entirely different from others is by no means a new observation. All writers on dietetics, popular or professional, have laid stress on the point, and have tried to explain these vagaries in a scientific or credulous spirit. In the Indian Vedas, where regimen and dietetics form by far the greater part of the prescribed treatment of the various described disorders, more or less elaborate rules are laid down for the administration of certain foods at certain seasons and the withholding of others at periods when, for unexplainable reasons, their exhibition would be detrimental to the digestion or health of the individual. The Salernian school extended and in some degree improved on these purely empirical principles, and later authors gave instances from their own experience or from hearsay to clinch the conclusions of these authorities. Enthusiastic reformers like Cornaro detailed their own individual experiences, which are worth more than the mass of theoretical assumptions. In modern times nearly every practitioner has had occasion to find out for himself examples of such individual idiosyncratic anomalies, some of which are of great interest. In many patients, for example, the eating of shellfish of any kind produces an erythematous rash, which has been mistaken for scarlet fever; of parsnips, a violent colic, attended with tympanites and actual tenderness over the abdomen, which has raised the suspicion of acute intestinal trouble. Urticarial rashes following the eating of common foods, such as Stilton cheese, scrambled eggs, and pea soup, are not infrequent, and every practitioner can recall one or more cases where a harmless (to an ordinary person) article of diet has led to symptoms which necessitated a medical examination. In the writer's own experience a perfectly normal patient invariably suffers from most distressing headaches after partaking of any dish into the composition of which asparagus enters. These cephalalgic attacks are accompanied with a transient urticarial rash, but rapidly pass off, apparently with the excretion of the well-known volatile constituent of this vegetable by the kidneys. Where such idiosyncrasies are known they rarely lead to mistakes in diagnosis or treatment, just as in the case of therapeutical idiosyncrasies, where the knowledge that a patient reacts abnormally to copaiba will prevent the medical attendant from diagnosing the interesting abdominal condition that sometimes results in such a case as one of peritonitis or intestinal obstruction. Yet they are matters for which the attendant should ever be looking out, and they are of relatively more importance in the event of a special diet or regimen being prescribed.

We are apt to assume that the dietetic value of a food is regulated by its physiological value—that is to say, that provided it contains the requisite amount of carbon, water, and nitrogen, is pleasant to the taste, and has a comparatively low digestive figure, it is an excellent food. So under normal conditions, to a physiologically normal individual, it would be; but here, as elsewhere, individual idiosyncrasy, in some cases at least, may play a great part. Digestive values are averages, and those obtained from experiments *in vitro* must be largely discounted in actual practice, where the individual peculiarities of the patient must be carefully studied. A case in point is found in white meats, which are popularly supposed to be far more easily digestible than red meats, and which, for that reason, are largely prescribed to convalescents. *In vitro* the digestive figure of very finely minced boiled chicken is not lower than that of equally finely minced boiled sirloin of beef, while it is certainly higher than that of minced raw sirloin. As usually served, unminced, boiled chicken is not more easily digestible than an underdone steak; the difference in digestibility will depend largely upon the amount of mastication. Thus, if the chicken were imperfectly masticated and imperfectly mixed with the saliva, while the steak was carefully chewed and well triturated with the digestive juices of the salivary glands, the former would be digested long after the latter. Again, the digestive figure of a certain article of food will vary with the digestive power of the individual, and, excluding cases where this factor is obviously modified by disease, the latter will depend to a certain extent upon the dietetic idiosyncrasies of the patient. These idiosyncrasies can only be ascertained by careful study, such study as the family practitioner is far better able to pursue than the specialist who is called in on occasions or who only sees the patient for the first time. A common and important instance of this is seen in the idiosyncrasy as far as milk and milk foods are concerned which some individuals show. Such patients cannot easily digest milk. For some reason when they are placed on a milk diet they do far worse than when put upon soups or broths, and in their case the amount of milk should be cut down to the minimum, and extracts, vegetable broths, jellies, or, in exceptional cases, artificial nutrients substituted. An idiosyncrasy will sometimes be shown towards a food when exhibited by the mouth, whereas the same food will be tolerated when given in the shape of a nutrient enema, or *vice versa*.

A consideration of these various facts shows that it is often useless to lay down dietetic rules applicable to all cases. They invariably break down in practice, for sooner or later the table that served excellently for the one case will be found entirely unsuitable for another and in all respects entirely similar case. This is well seen in diabetic patients, a class which shows marked idiosyncrasies towards certain articles of diet; but it is also true of many other conditions.

